# Proteomics analysis of carcinogenesis in a rat model of mammary cancer induced by DMBA (7,12-dimethylbenz[a]anthracene)

**DOI:** 10.12688/f1000research.132524.1

**Published:** 2023-06-06

**Authors:** Dyah Ayu Oktavianie Ardhiana Pratama, Anggun Nur Cahyati, Ulayatul Kustiati, Andreas Bandang Hardian, Fajar Shodiq Permata

**Affiliations:** 1 Veterinary Pathology Laboratory, Faculty of Veterinary Medicine, Universitas Brawijaya, Malang, East Java, 65143, Indonesia; 2 Study Program of Veterinary Medicine, Faculty of Veterinary Medicine, Universitas Brawijaya, Malang, East Java, 65143, Indonesia; 3 Laboratory of Veterinary Pharmacology, Faculty of Veterinary Medicine, Universitas Brawijaya, Malang, East Java, 65143, Indonesia; 4 Faculty of Veterinary Medicine, Universitas Gadjah Mada, Yogyakarta, Special Region of Yogyakarta, Indonesia; 5 Veterinary Anatomy, Histology and Embryology Laboratory, Faculty of Veterinary Medicine, Universitas Brawijaya, Malang, East Java, 65143, Indonesia

**Keywords:** Mammary cancer, DMBA, proteomic, protein profile, histopathology alteration

## Abstract

**Background:** Mammary cancer, called breast cancer in humans, results from the abnormal growth of cells in the mammary glands that attack the surrounding tissue. The process of carcinogenesis at the molecular level can be monitored through the production of proteins as biomarkers for carcinogenesis. 7,12-Dimethylbenz[a]anthracene (DMBA) is a known carcinogenic compound. This study aimed to analyze the proteomic profile as critical data regarding DMBA-induced carcinogenesis in Sprague‒Dawley rats.

**Methods:** Experimental animals were divided into two groups: a treatment group given DMBA at a dose of 10 mg/kg (intramammary) at intervals of 48 hours for a total of 10 doses, and a negative control group that was not given any treatment. Measurement of the total protein concentration was carried out using a spectrophotometer, and the data were analyzed using a t-test, while the characterization of protein profiles was carried out based on molecular weight data using SDS‒PAGE. Mammary gland histopathology was evaluated by hematoxylin and eosin (H&E) staining.

**Results:** The results showed a significant (p<0.05) increase of 27% in the total protein concentration in the rat mammary cancer model. The results of proteomic characterization showed a protein profile containing proteins of 187, 169, 68, 64, 53, 41, 24, 18, and 14 kDa, which were suspected to be HER-2, Nischarin, COX-2, Albumine, Vimentin, ACTB, TNF, p16, and fatty acid binding protein 3 (FABP3), respectively. Histopathology of the mammary glands showed an irregular and indistinct arrangement of the alveoli and extensive epithelial cell proliferation from the surface to the lumen of the mammary ducts, and the mammary stroma showed the formation of new epithelial cells, which were cancer cells that spread to surrounding tissue.

**Conclusions:** The proteomic profile was strongly associated with morphological alterations in mammary carcinogenesis in a rat model of DMBA-induced mammary cancer.

## Introduction

Mammary cancer, called breast cancer in humans, results from the uncontrolled growth of cells in the mammary glands, the glandular ducts extending from the lobule to the nipple, and the surrounding connective tissue. Mammary cancer can spread outside the mammary gland through blood vessels and lymph vessels. ^1^ The incidence of mammary cancer is high in animals, with incidence rates of between 41 and 53% in female dogs and approximately 86% in female cats. ^2^ The uncontrolled spread of cancer cells, or metastasis, can lead to death. ^3^ According to global cancer data, breast cancer is the most commonly diagnosed cancer and the fifth leading cause of death in women. ^4^ 7,12-Dimethylbenz[a]anthracene (DMBA) is a carcinogenic substance with cytotoxic, mutagenic, immunosuppressive, and carcinogenic properties. ^5^ DNA mutations were previously analyzed *in vitro* and *in vivo* in DMBA-induced mice. This analysis showed that 99% of DNA is depurinated by one-electron oxidation and that the 12-methyl of DMBA binds to the N-7 of adenine or guanine at an adenine:guanine ratio of 1:4. This binding forms a carcinogen-DNA adduct that affects DNA replication, thereby affecting the expression of the encoded protein. ^6^

DNA damage caused by carcinogens leads to uncontrolled gene mutations that result in protein changes and dysregulated cell proliferation, triggering the overgrowth of cancer cells. ^7^ Proteins are functional components of cells that regulate all cellular activities. Therefore, changes in protein expression in human breast cancer tissue play an important role in carcinogenesis. Malignant transformation at the molecular level can be detected through the excessive production of proteins encoded by oncogenes, namely, genes that stimulate cell growth and differentiation. Breast cancer refers to a group of neoplastic diseases with high intratumoral and intertumoral heterogeneity. Proteomic characterization of breast cancer has been used to understand molecular abnormalities, especially based on the signature of cancer-associated proteins (CAPs), a distinct group of potential cancer-associated biomarkers. Downregulation or upregulation of protein expression may contribute to the dysregulation of cellular functions, leading to tumor development and effects on patient survival. ^8^ For example, TNF-α, after binding to TNFR1 on the plasma membrane, activates NF-κB to promote the survival of tumor cells. Poor patient survival is associated with higher expression of TNF-α because TNF-α is a proinflammatory cytokine whose expression increases during tumor progression. ^9^ Overexpression of HER-2 in breast cancer can regulate antiapoptotic signals. ^8^

Based on this background, this study aimed to characterize the proteomic profile associated with histopathological changes in mammary cancer and can be considered a preliminary study identifying the molecular weights of the proteins expressed in mammary cancer tissue. In addition, the difference between the protein concentration in mammary cancer tissue and that in normal tissue was determined.

## Methods

### Research design

This study used rats as experimental animals obtained from and treated at Universitas Brawijaya. Rats were housed and maintained in a temperature-controlled room with a temperature of 21°C, a relative humidity of 45–65% and a 12 h/12 h light/dark cycle, with one week of acclimatization. Rats were provided food and water *ad libitum* during the treatment. Researchers and laboratory staff conducted observations twice a day. The experimental animals selected were 18 female Sprague–Dawley rats ( *Rattus norvegicus*), 10 weeks old, weighing 150–170 gr. The sample size was calculated using Federer’s formula:

$$\left(T-1\right)\;\left(N-1\right)>15$$

T = number of groups

N = number of samples.

This study was performed in accordance with the Animal Research: Reporting of *In Vivo* Experiments (ARRIVE) guidelines. ^34^ All treatments were carried out using the Three Rs principle based on the ARRIVE guidelines. The research was conducted in the laboratory of experimental animals, Faculty of Veterinary Medicine and Biosciences Laboratory, Universitas Brawijaya. Experimental animals were grouped into the control and treatment groups in a completely randomized design (CRD). GraphPad Prism (RRID:SCR_002798) version 8.0 was used to generate the CRD so that all samples received the same treatment. In this study, the number of necessary experimental animals was calculated using Federer’s formula to reduce the number of animals used. In this study, we only used healthy animals, both physically and physiologically, so animals that did not meet this requirement were excluded from the experiment.

### Ethical statement

We followed the standard ethical guidelines for animal care and use in this study. Moreover, all of the experimental protocols were approved by the Research Ethics Committee of the Universitas Brawijaya, Indonesia (Ethical Clearance No. 079-KEP-UB, approved on July 8 ^th^, 2021). The natural habits of the animals were considered, and we placed the animals under appropriate conditions as described above so that any harm, including pain and distress, to those animals was minimized.

### Blinding

In this study, researchers and laboratory staff were blinded to the identification numbers of the rats before their assignment to the control or treatment group in this study and remained blinded to those numbers throughout the study. The distribution of animals into the control and treatment groups was carried out using a CRD by laboratory staff who did not otherwise participate in this study to ensure there was no trend in group assignment and to prevent false-positive results.

### Animal model procedures

DMBA was dissolved in corn oil and normal saline (NS) at a DMBA:corn oil/NS ratio of 3:1. Tumorigenesis was induced in each rat in the treatment group by intramammary injection of DMBA at a dose of 10 mg/kg body weight (BW) in a 1 ml volume per rat, with 0.5 ml injected into the right and 0.5 ml injected into the left flank. Ten rounds of induction were conducted at 48-hour intervals. The tumors in rats in the treatment group were palpated 1-2 times a week after DMBA injection. The size of visible tumors did not exceed 40 mm, and the formation of only one tumor was induced, based on the guidelines of the Institutional Animal Care and Use Committee (IACUC). The rats in the control group did not receive any injection into the mammary glands. All rats were sacrificed by cervical dislocation, and the nodule that had formed was then excised from each rat in the treatment group, while the mammary gland was excised from each rat in the negative control group. The samples obtained were subjected to hematoxylin and eosin staining to describe the mammary gland structure, and proteomic profiling was carried out using SDS–PAGE. This study directly compared protein profiles and mammary gland tissues between normal rats and rats induced with DMBA.

### Histopathological examination

Formalin-fixed samples were gradually dehydrated in increasing ethanol concentrations and then cleared in xylene. Then, samples were embedded in paraffin and cut into five-micrometer sections using a rotary microtome. A standard hematoxylin and eosin (H&E) procedure was used to stain the sections, and images of the slides were acquired under a light microscope.

### Protein extraction

All samples were ground to homogeneity using a mortar. Then, 1.5 ml of PBST-PMSF was added to the extract. The suspension was sonicated for 20 minutes and then centrifuged at 6,000 rpm and 25°C for 15 minutes. The supernatant was transferred into a new 500 μl microtube, homogenized by adding absolute ethanol at an ethanol:suspension ratio of 1:1, and stored overnight in the freezer. Next, the sample was centrifuged at 10,000 rpm and 4°C for 15 minutes. The absolute ethanol was discarded, and the extract was air-dried until the odor of ethanol was no longer detectable and was then homogenized by adding 100 μl of Tris-HCl (pH 6.8). The extracts were stored at -20°C.

### NanoDrop spectrophotometry

Protein extraction was followed by NanoDrop spectrophotometry to measure the concentration and purity of the proteins in the sample. The NanoDrop instrument determines the protein concentration by measuring the absorbance of the sample at 280 nm. Protein concentrations were quantified by comparing the protein concentrations in the control and treatment groups as percentages.

### SDS–PAGE

SDS–PAGE was conducted according to the Mini-PROTEAN ^®^ Tetra Cell (Bio-Rad Laboratories Inc. #cat number 1658005, Berkeley, California) instruction manual. The concentrations of the extracted total protein samples were equalized to the lowest concentration in any sample, i.e., 21.14 mg/ml, and proteins were then denatured by the addition of Reducing Sample Buffer (SDS, Tris-HCl (pH 6.8), 100% glycerol, mercaptoethanol, and bromophenol blue) at a 1:1 ratio. The samples were then heated at 95°C for 5 minutes. A 12.5% resolving gel and a 3% stacking gel were used. The gel was mounted on the clamping frame and the frame was lowered into the mini tank; then, running buffer was added to the inner compartment of the clamping frame until the compartment was full. Samples that had been heated with RSB were then loaded into each well at a volume of 20 μl, and one well was filled with protein marker. Electrophoresis was performed at 100 V until the tracking dye reached a level of 0.5 cm above the bottom of the resolving gel. The staining process began with the removal of the gel from the plate, and the gel was then placed in a container containing Coomassie blue stain. The gel was incubated with shaking for 10 minutes so that the dye was evenly distributed, and color removal of the remaining dye was performed by overnight incubation with a mixture of methanol, glacial acetic acid, and H _2_O in a shaker.

### Statistical analysis

The concentrations determined for the control and treatment groups were analyzed with IBM SPSS Statistics (RRID:SCR_016479) version 26 using the Shapiro–Wilk normality test and Levene’s test for homogeneity with a significance level of 0.05 (α = 0.05). Then, an independent samples t-test was conducted to analyze the difference in concentration between the groups. Two tests were performed with two replicates each. We visualized the bands on the SDS–PAGE gels and predicted the name of the protein corresponding to each band based on calculation of its molecular weight.

## Results

### Total protein concentration

The results of analysis of the average total protein concentrations in the two groups using an independent samples t-test are presented in Table 1. ^33^

**Table 1.** Total protein concentrations in the animal model. DMBA, 7,12-dimethylbenz [a]anthracene.

Group	Total protein concentration (mg/ml) ± SD	Improvement compared to control	Sig. (2-tailed)
Control	31.15 ± 7.40		0.006
DMBA-treated	39.65 ± 3.11	27%	

Significance analysis using the independent samples t-test showed a Sig (2-tailed) value of 0.006. These results indicated that the two groups had significantly different mean protein concentrations (p < 0.05). The total protein concentration in the treatment group was increased by 27% compared to that in the negative control group.

### Visualization of the protein profile in mammary glands

**Figure 1.**
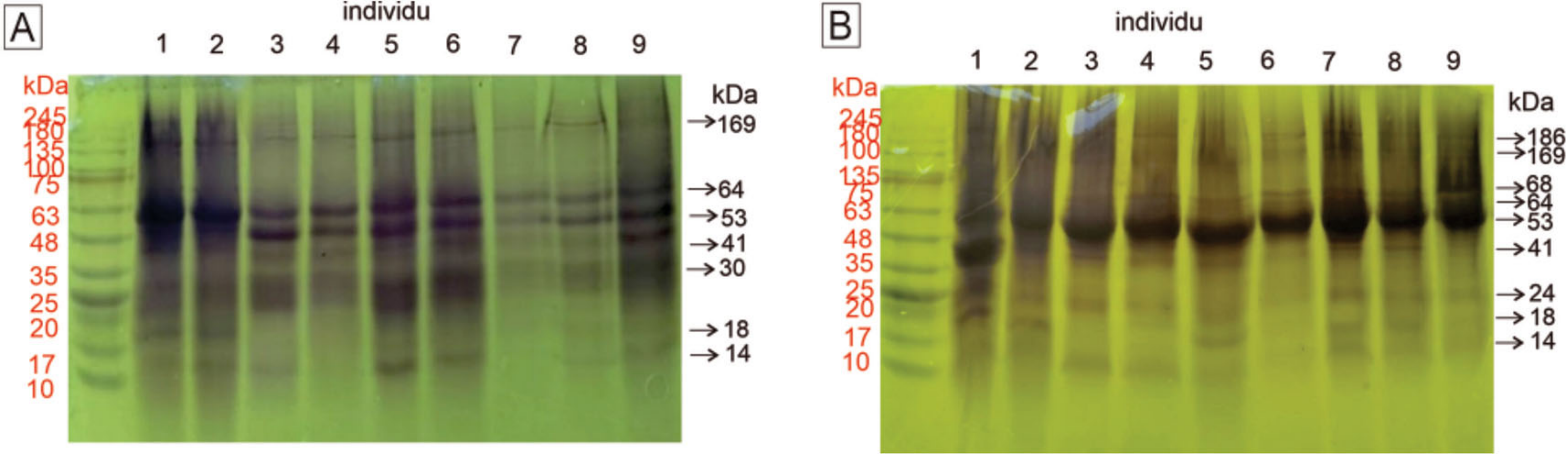
Visualization of the protein profiles of mammary gland samples by SDS–PAGE (12.5%). A. Control group. By gel electrophoresis, seven bands at 14, 18, 30, 41, 53, 64, and 269 kDa were observed in the control group. B. Nine bands at 14, 18, 24, 42, 53, 64, 68, 169, and 186 kDa were observed in the treatment group. Between the control and treatment groups, we observed three different bands. The proteins are described in Table 2. The molecular weights of the proteins in the molecular marker are shown in red, and the molecular weights of proteins in the samples are shown in black.

The visualization of the protein profile in rat mammary gland tissue samples determined using 12.5% SDS–PAGE is presented in Figure 1. The observable protein bands on the SDS–PAGE gel can describe the state of the carcinogenic process in the rat mammary gland. In addition to the appearance of a protein band, the thickness of the protein band indicates the concentration of the protein. The classification of proteins expressed in rat mammary gland tissue, as determined by electrophoresis, is shown in Table 2.

**Table 2.** Classification of proteins expressed in rat mammary tissue as visualized by SDS–PAGE. DMBA, 7,12-dimethylbenz [a]anthracene; FABP3, fatty acid binding protein 3.

Protein band	Molecular weight (kDa)		Presumptive protein identification & molecular weight (kDa)	Protein classification
	Control group	DMBA-treated group		
1	-	187	HER-2 (187) Nischarin (166)	Protooncogene-encoded protein
2	169	169		Tumor suppressor protein
3	-	68-71	COX-2 (68)	Inflammatory mediator
4	64	64	Albumine (65)	Plasma protein
5	53-55	53-55	Vimentin (53)	Filament protein
6	41-43	41-43	β-Actin (41)	Filament protein
7	30	-	Prohibitin (29)	Tumor suppressor protein
8	-	22-26	TNF-α (25)	Inflammatory mediator
9	18	18-20	p16 (17)	Tumor suppressor protein
10	13-14	11-14	FABP3 (14)	Tumor suppressor protein

### Histopathological examination

**Figure 2.**
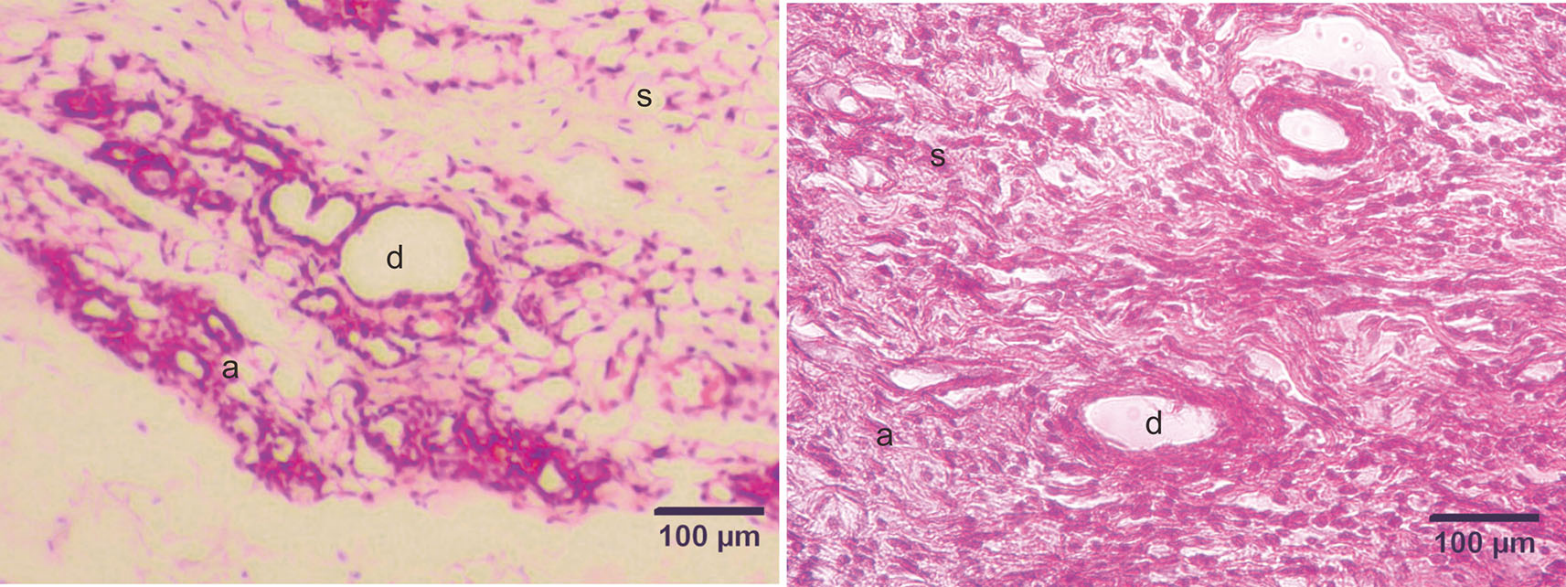
Micrograph of the mammary stroma in mammary tissue from control group rats and DMBA treatment group rats, with hematoxylin and eosin staining. A. Control group. B. Treatment group. The mammary stroma showed the formation of new epithelial cells that were cancer cells and spread to surrounding tissues and hyperplasia with mitotic fibrocytes in the dermis. (a) alveoli; (d) ducts; (s) stroma. Scale bar, 100 μm. DMBA, 7,12-dimethylbenz [a]anthracene.

**Figure 3.**
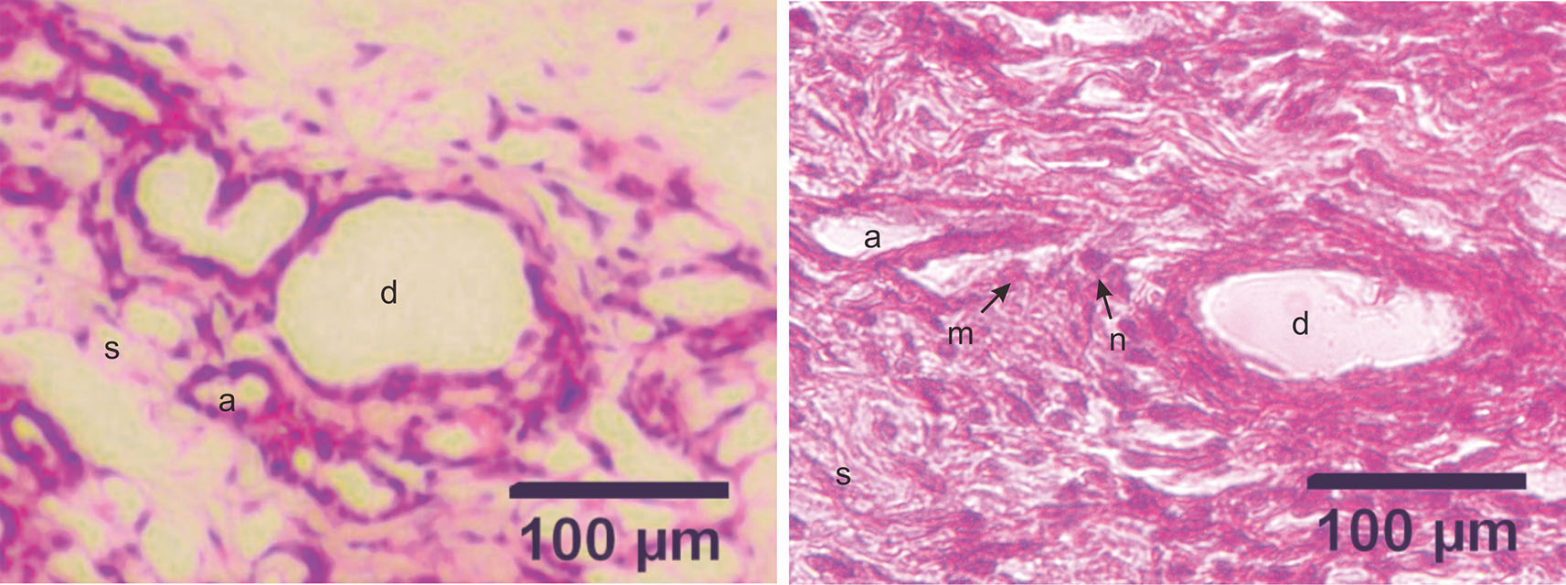
Micrograph of alveoli and mammary ducts in mammary tissue from control group rats and DMBA treatment group rats, with hematoxylin and eosin staining. A. Control group. The alveoli contain irregular and cuboid cells with no hyperchromatic nuclei. B. Treatment group. The alveoli in the treatment group showed an irregular and indistinct arrangement. The epithelial cells were not cuboidal and contained hyperchromatic nuclei. The mammary ducts were filled with proliferating epithelial cells from the surface of the ducts to the lumen of the ducts. (a) alveoli; (d) ducts; (s) stroma; (m) malignant cells (the cells appeared larger); (n) nuclei (hyperchromatic with a large size). Scale bar, 100 μm. DMBA, 7,12-dimethylbenz[a]anthracene.

The development of DMBA-induced tumors in the mammary glands of rats in the mammary cancer model was observed through H&E staining. The histology of the mammary glands under normal conditions (control group) in this study showed that the alveoli exhibited a rounded structure with a surface lined with epithelial cells, myoepithelial cells, and basal membranes. The mammary ducts were empty and lined with cuboidal epithelial cells. The stroma comprises connective and fat tissue ( Figure 2). Histopathological examination of the mammary glands of rats in the model of DMBA-induced mammary cancer (treatment group) showed an irregular and indistinct arrangement of the alveoli. The epithelial cells were not cuboidal and contained hyperchromatic nuclei. The mammary ducts were filled with proliferating epithelial cells from the surface of the ducts to the lumen of the ducts. The mammary stroma showed the formation of new epithelial cells that were cancer cells and spread to surrounding tissues ( Figure 3).

## Discussion

In the present study, mammary cancer development in DMBA-induced rats was confirmed by proteomic analysis and histopathological examination of the mammary glands of rats. The total protein concentration in the DMBA-treated rats was increased by 27% compared to that in the control group. This finding is also consistent with a previous study, in which the total Albumine and globulin concentration in a group of female patients with breast cancer had an average value of 7.63 ± 0.14 g/dl and was higher than those in the control group and in the group of healthy women, in which the mean value was 6.14 ± 1.84 g/dl. ^10^ In this study, the difference between the protein concentration in the control group and the DMBA-treated group of rats was thought to be related to proto-oncogenes that play a role in promoting and increasing cell growth and division, which can become activated if there is an alteration or mutation. The increased appearance of oncoprotein bands is a characteristic related to excessive oncoprotein expression in cells. There is an increase in the production of mRNA transcripts of oncogenes and in the number of copies of oncogenes. ^11^ The carcinogenesis process at the molecular level can be detected by the excessive production of oncoproteins. Activation of oncogenes stimulates faulty production of growth factor receptors, indicating continued growth in the absence of external stimuli. Uncontrolled cell division without cell maturation causes impaired cell differentiation. ^12^ At a later stage, impaired cell differentiation causes malignant transformation of cells. In this study, exposure to DMBA as a carcinogenic agent caused changes in the nucleotide sequence of proto-oncogene DNA, resulting in changes in gene expression. ^13^

Proteomic profiling was performed using SDS–PAGE to visualize changes in gene expression in DMBA-treated rats. The observable protein profile can describe the state of carcinogenesis in rat mammary gland tissue. In addition to the appearance of a protein band, the thickness of the protein band indicates the protein concentration. According to the results of this study, a protein with a molecular weight of 187 kDa was expressed in the DMBA-treated group but not in the control group. This protein is suspected to be the HER-2 protein, which has a molecular weight of 185 kDa ( https://www.uniprot.org/uniprotkb/Q003I6/entry). ^14^ HER-2 is a proto-oncogene that controls the growth and development of mammary epithelial cells. In mammary carcinomas in rats, there is amplification of this gene such that the cell division rate is doubled and cell growth increases. ^15^ DNA damage due to DMBA exposure results in a single-amino acid mutation by substituting a valine residue in the transmembrane domain with glutamic acid. ^16^ This substitution induces dimerization of the HER-2 oncoprotein in the cell membrane, with results similar to the induction of activation by growth factors so that activation continues even in the absence of external growth stimuli. ^17^ Overexpression of the HER-2 gene occurs in approximately 15–30% of breast cancers and correlates with cell proliferation. ^18^ This finding relates to the histopathological appearance shown in Figure 1B, which was characterized by cell proliferation in the alveoli and from the surface to the lumen of the mammary ducts.

A protein band with a molecular weight of 169 kDa was visible in all tissues, in both the negative control and DMBA-treated groups, and there was no difference in the protein band thickness between the two groups. The corresponding protein was considered to be Nischarin, with a molecular weight of 166 kDa ( https://www.uniprot.org/uniprotkb/A0A8I5ZTG9/entry). Nischarin is an integrin-binding protein known to be a novel tumor suppressor and to play a critical role in mammary cancer cell migration and invasion in rats. ^19^ Research conducted by Chang *et al.* ^20^ showed that increased Nischarin expression in tumor cells reduces the invasive and migratory abilities of cancer cells. Analysis of a total of 962 patients with breast cancer revealed that 0.3% of invasive carcinomas showed loss of Nischarin expression and that 0.7% of invasive carcinomas expressed mutated Nischarin. ^21^

A protein band with a molecular weight of 68 kDa was observed in all mammary gland samples of DMBA-treated rats and was not observed in samples from negative control rats. The corresponding protein was suspected to be the COX-2 protein, with a molecular weight of 69 kDa ( https://www.uniprot.org/uniprotkb/Q8CIP1/entry). COX-2 plays a role in the conversion of arachidonic acid to prostaglandins. COX-2 induces angiogenesis through three products, thromboxane A2, prostacyclin, and prostaglandin E2, which stimulate vascular endothelial growth factor (VEGF) production in angiogenesis. COX-2 upregulation is observed in 40% of patients with invasive breast carcinoma and correlates with poor prognosis. COX-2 upregulation promotes tumor formation and progression to metastasis, in addition to increases in angiogenesis, cell migration, and invasion. ^22^ This finding can be related to the histopathological image shown in Figure 3B, which shows the formation of new epithelial cells in the mammary stroma that were cancer cells spreading to the surrounding tissues.

A protein band with a molecular weight of 64 kDa was observed in all mammary cancer tissues and all normal tissues, but there was a difference in the appearance of the bands formed. The band visualized in the negative control sample was thicker than the band visualized in the treatment group sample. This indicates a reduction in the concentration of the protein in the treatment group sample. The corresponding protein was thought to be Albumine, with a molecular weight of 65 kDa ( https://www.uniprot.org/uniprotkb/A0A0G2JSH5/entry). Cancer and lack of nutrition are among the causes of a decreased Albumine level. Cancer cells proliferate over an indefinite period of time, and the number continues to increase. Under these conditions, cancer cells and normal cells compete for nutrients. Cancer cells require all available nutrients, resulting in the death of normal cells due to a lack of nutrients.

A protein band with a molecular weight of 53 kDa was observed in all negative and DMBA-treated samples. There was a thickening of the band in all treatment group samples. The corresponding protein was suspected to be Vimentin, which has a molecular weight of 53 kDa ( https://www.uniprot.org/uniprotkb/P31000/entry). Vimentin is a protein that forms intermediate filaments, which are components of the cytoskeleton, and is expressed in cells that are embryonically derived from the mesenchyme and in epithelial cells. ^23^ Vimentin is used as a marker of mesenchymal cells or cells undergoing epithelial-to-mesenchymal transition (EMT) during normal and metastatic development. ^24^

In both groups, a 41 kDa protein band was visible, but in the DMBA-treated group, the band was thickened. This protein was predicted to be actin (ACTB), which has a molecular weight of 41 kDa ( https://www.uniprot.org/uniprotkb/Q9JLX9/entry). ACTB plays important roles in cell migration and division. Analysis of ACTB expression based on The Cancer Genome Atlas (TCGA) database showed abnormal expression of ACTB in breast cancer cases with mutation or amplification of ACTB. That study also showed that ACTB expression in invasive breast carcinoma tissue was higher than that in normal tissue and that there was a relationship between high ACTB expression and poor prognosis in breast cancer. ^25^

Negative control samples showed a protein band with a molecular weight of 30 kDa that was not observed in DBMA-treated samples. The corresponding protein was thought to be prohibitin, which weighs 29 kDa ( https://www.uniprot.org/uniprotkb/Q5XIH7/entry). Prohibitin can inhibit cell proliferation by inducing blockade of the S/G2 transition and promoting apoptosis. ^26^ A study conducted by Najm *et al.* ^27^ in cancers showed a missense mutation located in the sequence of the prohibitin gene encoding the Rb-binding domain, causing the production of abnormal protein products and loss of activity.

The protein band with a molecular weight of 24 kDa was observed only in the DMBA-treated group. The corresponding protein was predicted to be TNF-α, with a molecular weight of 25 kDa ( https://www.uniprot.org/uniprotkb/P16599/entry). In a mammary cancer model, rats with mammary cancer showed greater proinflammatory cytokine secretion than normal rats. This is thought to be due to an enhanced immune response associated with increased immune cell activity due to the presence of antigens. Cancer cells express antigens in the form of mutated genes or overexpressed normal proteins. These antigens are recognized by the immune system to elicit an immune response. ^28^

The protein band showing a molecular weight of 18 kDa was observed in all treatment group samples except for K3 and K4. This protein was considered to be p16 in the INK4 family, with a molecular weight of 17 kDa ( https://www.uniprot.org/uniprotkb/Q9R0Z3/entry). The INK family is a family of cyclin-dependent kinase inhibitors that can inhibit Cdk activity by forming a stable complex with Cdk proteins, thereby preventing Cdk proteins from binding to cyclin D during the cell cycle. Deregulation of the cell cycle is a crucial feature of carcinogenesis. P16 is one of the proteins involved in the cell cycle. The expression of p16 in cancer can constitutively inactivate the tumor suppressor retinoblastoma (Rb). The change in p16 expression leads to gene downregulation and promotes the formation of epithelial cancers. ^29^ In this study, seven treatment group samples expressed the p16 protein, while the other two did not, a phenomenon thought to indicate the heterogeneity of this type of cancer. An association with p16 overexpression was found for breast cancer, and p16 expression was found to be a prognostic indicator in breast cancer. ^29^ In both of these cases, the luminal A subtype showed lower p16 expression than the other subtypes. In contrast, the triple-negative subtype showed higher p16 expression than the other subtypes. Some cancers occur due to the complete loss or malfunction of tumor suppressor genes. According to Musyarifah *et al.*, ^30^ positive p16 expression is more likely to be present in cancers with higher stages and higher histopathological degrees.

Regarding the protein band showing a molecular weight of 14 kDa, observed in the negative and treatment groups, the corresponding protein was suspected to be fatty acid binding protein 3 (FABP3), with a molecular weight of 14 kDa ( https://www.uniprot.org/uniprotkb/A0A8I6A2E9/entry). FABP3 plays roles in fatty acid transport, cell growth, and gene transcription. In cancer, this protein acts as a tumor suppressor. ^31^ Reduced FABP3 expression in breast cancer cells was confirmed to occur by hypermethylation of the FABP3 promoter. ^32^ Hypermethylation is an increase in methylation in DNA that should not be methylated. Inhibition of transcription due to DNA methylation in the promoter prevents the gene from being expressed. Research on the molecular characteristics of mammary cancer as markers of cancer incidence has provided new information regarding the molecular heterogeneity of tumors. Molecular characterization has revolutionized mammary cancer research and the choice of therapy. Because mammary cancer in animals or breast cancer in humans is a disease with a high mortality rate, consideration of molecular profiling is anticipated to be very important in the selection of treatment options and development of new drugs.

## Conclusions

The total protein concentration in the mammary tissues of rats with DMBA-induced mammary cancer was significantly increased by 27%. The proteins determined by SDS–PAGE to be expressed in mammary tissues in the rat model of DMBA-induced mammary cancer were thought to be HER-2, Nischarin, COX-2, Albumine, Vimentin, Actin, TNF-α, p16, and FABP3. DMBA induction can activate the expression of mammary cancer marker proteins, such as HER-2 and COX-2, and reduce the expression of the prohibitin protein, which plays a role in mammary carcinogenesis. The protein profile of mammary tissue was associated with its histopathological appearance, which showed an irregular and indistinct arrangement of the alveoli, proliferation of mammary duct epithelial cells, and the formation of new epithelial cells in the mammary stroma. In this study, we found that proteins with altered expression in cancer can be markers of tumorigenesis, diagnostic and prognostic markers, and therapeutic targets. This study discusses protein expression in an animal model of DMBA-induced mammary cancer. We found several potential protein biomarkers related to breast cancer development pathways. We hope these findings will encourage further clinical validation studies and provide insights and directions for future research.

## Data availability

### Underlying data

Zenodo: The Role of Proteomics Analysis in Carcinogenesis in a Rat Mammary Cancer Model Induced by DMBA (7,12-dimethylbenz(α)anthracene). https://doi.org/10.5281/zenodo.7796773. ^33^

This project contains the following underlying data:

- Protein Molecular Weight.xlsx
- Protein Nanodrop Spectrophotometer.xlsx

### Reporting guidelines

Zenodo: ARRIVE checklist for ‘The role of proteomics analysis in carcinogenesis in a rat mammary cancer model induced by DMBA (7,12-Dimethylbenz[a]anthracene)’. https://doi.org/10.5281/zenodo.7839767. ^34^

Data are available under the terms of the Creative Commons Attribution 4.0 International license (CC-BY 4.0).
