# Supplementary material for: Proteomics analysis of carcinogenesis in a rat model of mammary cancer induced by DMBA (7,12-dimethylbenz[a]anthracene)
Source: F1000Res. 2024 Aug 5;12:606. Originally published 2023 Jun 6. [Version 2] doi: 10.12688/f1000research.132524.2 (PMC11282392; doi:10.12688/f1000research.132524.2)
Supplement: Peer review history

# Reviewer report: Reviewer response for version 1

**DOI:** 10.5256/f1000research.145453.r305378

**Published:** 2024-08-05

**Authors:** Jasni Bin Sabri

**Affiliations:** 1 Universiti Malaysia Kelantan, Pengkalan Chepa, Kelantan, Malaysia; 2 Faculty of Veterinary Medicine, Universitas Brawijaya, Malang, East Java, Indonesia

This is a very good study and has present and future worldwide applications for cancer prognosis and diagnosis that can assist decision making for cancer patient management. 

  I have a few comments that can be useful to improve the article;

 1. The title is good. However, an alternative title is applicable and can be more meaningful as suggested below: " Proteomic Analysis of Mammary Cancer Cells Carcinogenesis Induced by DMBA (7,12-dimethylbenc[a] in Sprague-Dawley Rats"

 Since there are many species of rats, specifically indicating the types of rats used in this study in the title is reasonable. Different rats may respond differently to research methodology and the related materials used.

 2. Abstract

 a) Background

 Line 2, the word "attack" is less scientific and do not much reflect a pathology term (layman). It is better to change it to "infiltrate"...to better define the usual cellular feature of neoplastic cancer growth.

 b) Methods: I believe there is no evaluation conducted on histopathological examination (no lesion scoring). The histopathology is only descriptive to support and validate that the proteomic analysis is associated with the tumor successfully induced in the rats.

 Therefore the word "evaluated" (last line) should be replaced with " examined".

 c) Results: Line 6: the words "..........from the surface to the lumen of mammary duct,...." is unclear. Is this referring to epithelial cells lining the duct that proliferated from the basement membrane (or from the epithelial lining to the basement membrane), to the surface, that is towards the lumen of the duct? The sentence need to be reconstructed for clarity.

 3. Introduction: Sentence No 2, Line 3, "to spread outside the mammary gland": It is better to change these words to "can spread to other organs and tissues". I believe these words are more clearer and meaningful.

 3. Histopathology Image (Figure 2)

 a) The two images need to be labelled; Which one is A and which one is B? Left or Right? This will help any reader of this article to easily and correctly refer and relate between the details of both images.

 b) There is also no cellular description for the image of the control group rat. Since the images of both groups of rats are shown, the description of both, is necessary.

 c) The word "hyperplasia" is not accurate for cellular description for tumor (neoplasia). The word hyperplasia is mitosis (cellular division) of normal cells that the body cell proliferation signaling system can control. In contrary, for neoplasia, it is an uncontrolled cellular proliferation associated with carcinogens that stimulates the cellular growth. However, I believed, the hyperplasia in the figure is referring to the fibrocytes. It is also important to describe the mitotic index (high or low) of the mammary cancer cells in this study.

 d) The cellular description for the rat in the treatment group can be improved. Importantly, to include the description that strongly suggests the cellular features of malignant neoplasia (cancer), such as pleomorphism (variation in cell sizes and shapes), the mitotic index (high or low) etc. If for instance, neoplastic cells are seen present in the lumen of blood vessels and/or lymphatic vessels (tumor emboli), it would be better because this validate the tumor is malignant, knowing that, only malignant tumors can spread to other body sites (ie: an indication of not a benign tumor). However, seeing these evidences, are of rare occasion and can be exhaustive. Other supportive cellular features of malignancy (pleomorphism, high mitotic index etc.), are acceptable.

 d) Figure 3: Which one is the control and Treated group? The left or right image? Labeling is required. 

 The legend for Figure 3 needs a more clearer description. 

 For B, the treated group rat, if the cells are not cuboidal? What are the changes in shape when neoplastic? Describe the changes. 

 This is my opinion for the description of the duct in B as follows;

 The duct in the treated group rat is thickened and appeared eosinophilic with less discernable nuclei. The thickened duct shows reduction in size of its lumen.

 The duct in the control rat is single layered with visible nuclei of the epithelial cells lining the duct.

 e) Conclusion

 Second sentence, line 3; The words "......were thought to be...."  can be replaced with better words like, " suggestive of " or "likely".

 This conclusion is based on molecular weight data analyses between the protein profiles (proteomic) of the current work and referring to previous studies.

Is the work clearly and accurately presented and does it cite the current literature?

Partly

If applicable, is the statistical analysis and its interpretation appropriate?

Yes

Are all the source data underlying the results available to ensure full reproducibility?

Yes

Is the study design appropriate and is the work technically sound?

Yes

Are the conclusions drawn adequately supported by the results?

Yes

Are sufficient details of methods and analysis provided to allow replication by others?

Yes

Reviewer Expertise:

Pathology: Pathogenesis, Histopathology and Ultrastructural Pathology.

I confirm that I have read this submission and believe that I have an appropriate level of expertise to confirm that it is of an acceptable scientific standard.

# Reviewer report: Reviewer response for version 1

**DOI:** 10.5256/f1000research.145453.r181616

**Published:** 2024-07-26

**Authors:** Hevi Wihadmadyatami

**Affiliations:** 1 Department of Anatomy, Faculty of Veterinary Medicine, Universitas Gadjah Mada, Yogyakarta, Indonesia

Introduction: Please make a stronger background on the importance of the detection of the cancer biomarker or proteomics analysis on the rat model.

 Material and method: Please complete all the data of the reagent which is used on the experiment.

 Results: Please provide a more clean version on the part of molecular weight of figure 1, so the reader can read clearly the molecular weight of each protein which is expressed on the mammary tissue gland.

Is the work clearly and accurately presented and does it cite the current literature?

Yes

If applicable, is the statistical analysis and its interpretation appropriate?

Yes

Are all the source data underlying the results available to ensure full reproducibility?

Yes

Is the study design appropriate and is the work technically sound?

Yes

Are the conclusions drawn adequately supported by the results?

Yes

Are sufficient details of methods and analysis provided to allow replication by others?

Yes

Reviewer Expertise:

Cell biology, cancer mechanism, neurodegeneration, histology

I confirm that I have read this submission and believe that I have an appropriate level of expertise to confirm that it is of an acceptable scientific standard.

# Response

**Published:** 2024-07-29

**Authors:** Dyah Ayu Pratama

**Affiliations:** Faculty of Veterinary Medicine, Universitas Brawijaya, Malang, East Java, Indonesia

Dear Reviewer,

 I appreciate and am thankful for your positive comments and suggestions. 

 As your suggestion on introduction section, I have highlight the importance of this research especially related with proteomic analysis of cancer biomarkers on cancer detection and prediction, as well in the development of cancer therapy.

 I have also complete the data of reagent which is used in this research.

# Reviewer report: Reviewer response for version 1

**DOI:** 10.5256/f1000research.145453.r181612

**Published:** 2024-07-26

**Authors:** Muhammad Luqman Nordin

**Affiliations:** 1 Department of Veterinary Clinical Studies, Faculty of Veterinary Medicine, Universiti Malaysia Kelantan, Kota Bharu, Malaysia

Overall, this article is good and contributes to the novelty and very valuable knowledge especially with regards to mammary carcinoma in animals. As the prevalence of cancer is increasing among animals, this study provides a high level of academic and scientific writing.

 Minor comment: it would be better if you can provide a picture of mammary cancer development on rats. The mammary cancer results from the uncontrolled growth of cells in the mammary glands, the glandular ducts extending from the lobule to the nipple, and the surrounding connective tissue. Normally, there will be masses present at the nipple/mammary area.

Is the work clearly and accurately presented and does it cite the current literature?

Yes

If applicable, is the statistical analysis and its interpretation appropriate?

Yes

Are all the source data underlying the results available to ensure full reproducibility?

Yes

Is the study design appropriate and is the work technically sound?

Yes

Are the conclusions drawn adequately supported by the results?

Yes

Are sufficient details of methods and analysis provided to allow replication by others?

Yes

Reviewer Expertise:

veterinary oncology, pharmacology, toxicology and pharmaceutics ( vaccine and drugs delivery technology).

I confirm that I have read this submission and believe that I have an appropriate level of expertise to confirm that it is of an acceptable scientific standard.

# Response

**Published:** 2024-07-29

**Authors:** Dyah Ayu Pratama

**Affiliations:** Faculty of Veterinary Medicine, Universitas Brawijaya, Malang, East Java, Indonesia

Dear Reviewer, 

  Thank you very much for your positive comments and suggestions. Related with your suggestion to provide a description of mammary cancer development on rats, I have supplemented with an explanation of the development of mammary cancer in rats as a result of DMBA induction.
